# How to Do a Bedside Methylene Blue Nephrostogram to Confirm Ureteral Patency Following PCNL

**DOI:** 10.1111/ans.70271

**Published:** 2025-07-25

**Authors:** Paul M. Rival, Yajat Dua, Niranjan Sathianathen, Simeon Ngweso, Briony Norris, Shomik Sengupta

**Affiliations:** ^1^ Department of Urology Eastern Health Melbourne Victoria Australia; ^2^ Monash University Melbourne Victoria Australia

**Keywords:** methylene blue, nephrostogram, PCNL, ureteral patency

## Abstract

This article introduces the bedside methylene blue nephrostogram (BMBN) as a rapid, cost‐effective alternative to traditional imaging for confirming ureteral patency post‐percutaneous nephrolithotomy (PCNL). The study details the procedure, supported by case studies, demonstrating its potential to expedite patient discharge, reduce hospital stays, and alleviate healthcare costs in Australian public hospitals.

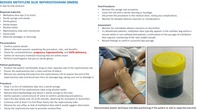

## 1 Introduction

With growing pressures on public hospitals in Australia and the ever‐increasing need for bed availability, the prompt clinical review and safe discharge of post‐operative patients is crucial. Following percutaneous nephrolithotomy (PCNL), the wait for imaging services to confirm ureteral patency before nephrostomy tube removal often extends hospital stays and escalates healthcare costs. In this context, the introduction of a bedside methylene blue nephrostogram (BMBN) presents a promising alternative by confirming ureteral patency without the need for further imaging. This article explores the utility and advantages of BMBN in post‐PCNL patient management.

## 2 Contra‐Indications

Documented contraindications include pregnancy, hypersensitivity reactions, and G6PD deficiency. Anaphylactic shock is rare; however, monitoring while injecting and immediate cessation of administration are still advised if symptoms arise.

## 3 Step‐by‐Step Instructions

### 3.1 Pre‐Procedure

Gather the required materials, ensuring that methylene blue dye, a sterile syringe with needle, sterile gloves, drapes, alcohol swabs, a nephrostomy tube with connectors, gauze pads, and adhesive bandages or dressings are readily available within the procedural area. Begin by confirming the patient's identity and details. Obtain informed consent and check for any contraindications. Comfortably position the patient on their side, opposite the nephrostomy site, ensuring the area is clean and free from any debris. Remove any existing dressing from the nephrostomy site to expose the end of the nephrostomy tube and disconnect it from its drainage bag, taking care not to dislodge it. Perform hand hygiene and don sterile gloves.

### 3.2 Procedure

Using a sterile technique, draw 2 to 5 mL of methylene blue dye into the syringe. Clean the end of the nephrostomy tube using alcohol swabs and attach the prepared syringe to the tube. Confirm tube placement and patency by gently aspirating a small sample of urine. Slowly inject the methylene blue dye over a 10 to 30‐s duration, carefully observing for any signs of resistance or discomfort. Continue the injection until at least 2 to 5 mL of dye has freely entered the nephrostomy tube. Watch for any reflux or leakage of the dye, which may indicate an obstruction. Finish by clamping the nephrostomy tube to promote ureteral flow.

### 3.3 Post‐Procedure

After injecting the dye, disconnect the syringe and securely cover the nephrostomy site with a sterile dressing or bandage. Document the procedure in the patient's medical notes, noting any immediate complications.

### 3.4 Assessment

Monitor the patient for any immediate or delayed adverse reactions or complications. Confirm successful dye passage by recording the findings, which should be clear evidence of blue‐tinted discolored urine, signifying ureteral patency.

Methylene blue is a safe, cheap, easily available, and approved therapeutic product with multiple well‐documented clinical uses including its routine use during PCNL procedures [1, 2]. Recorded adverse effects include serotonin syndrome risk when combined with other serotonergic drugs, dizziness, confusion, headaches, and urine discoloration [1]. The “gold standard” antegrade nephrostogram (AN) to assess ureteral patency presents several limitations. First, it is a lengthy investigation, taking at least 90 min to perform when including patient transport times. Second, it exposes the patient to radiation and contrast agents known to potentially cause serious hypersensitivity reactions. Third, it requires interventional radiology. Fourthly, it is costly, with a rebatable cost of $164.20 (AUD) according to the Australian Medicare website [2]. Unlike ANs, BMBNs provide a rapid, low‐cost, accurate, and real‐time evaluation. With a 5 mL vial of methylene blue costing about $26 (AUD) and its rapid test‐to‐result time of approximately 20 min, it allows for a prompt clinical review and optimization of hospital resources [3]. Of note, in a prospective study of post‐PCNL patients, combining a capping trial with BMBN (AUC 0.72; 95% CI 0.61–0.84), as well as BMBN alone (AUC 0.71; 95% CI 0.60–0.83), achieved comparable accuracies to AN (AUC 0.78; 95% CI 0.68–0.88) in predicting successful nephrostomy tube removal, which supports their use in postoperative protocols with a preference for using a combined approach [4]. In conclusion, BMBN is a safe, tolerable, rapid, and cost‐effective procedure with no radiation exposure able to confirm ureteral patency following PCNL in patients with a nephrostomy tube.

## Author Contributions

**Paul M. Rival:** conceptualization, formal analysis, funding acquisition, investigation, methodology, project administration, writing – original draft, writing – review and editing. **Yajat Dua:** writing – original draft, writing – review and editing. **Niranjan Sathianathen:** conceptualization, supervision, writing – review and editing. **Simeon Ngweso:** conceptualization, methodology, supervision, writing – review and editing. **Briony Norris:** supervision, writing – review and editing. **Shomik Sengupta:** conceptualization, supervision, validation, writing – review and editing.

## Supporting information


**Data S1:** Supporting Information.

**Video S1:** Demonstration of a bedside methylene blue nephrostogram (BMBN).
