# Supplementary material for: How to Do a Bedside Methylene Blue Nephrostogram to Confirm Ureteral Patency Following PCNL
Source: ANZ J Surg. 2025 Jul 25;95(12):2610–1. doi: 10.1111/ans.70271 (PMC12717479; doi:10.1111/ans.70271)
Supplement: Supplementary file 1 — Data S1: Supporting Information. [file ANS-95-2610-s002.docx]

**How to do a bedside methylene blue nephrostogram to confirm ureteral patency following PCNL – *Supporting document.***

*Paul M Rival^1,2^, Yajat Dua^2^, Niranjan Sathianathen^1^, Simeon Ngweso^1^, Briony Norris^1^*, *Shomik Sengupta^1,2^*

**Affiliations**

^1^ Department of Urology, Eastern Health, Melbourne, Victoria, Australia

^2^ Monash University, Melbourne, Victoria, Australia

Corresponding author:

Paul Rival

Address: Level 2, 5 Arnold St, Box Hill, Victoria 3128, Australia

Email: paul.rival@monash.edu

Telephone: 0429817670

Discussion

As demonstrated in a previously published case report, BMBN to assess ureteral patency presents several noteworthy advantages and has the potential to improve care strategies for post-operative PCNL patients.^1^ The efficacy of the BMBN was also demonstrated through two cases at our centre. The first case featured a 49-year-old male with spina bifida and a complex urological history (Mitrofanoff and left-to-right Transureteroureterostomy) who presented to ED with renal colic and was found to have a left 8mm ureteric stone proximal to the L-R anastomosis. He underwent a supine PCNL and had a 10Fr nephrostomy tube inserted post-operatively. He was scheduled to have his nephrostomy tube removed if patency was confirmed on nephrostogram the following day. However, due to delays in the radiology department, he was still awaiting the nephrostogram the following evening and following an informed discussion with the Urology team, consent was given by the patient for a BMBN that successfully confirmed ureteral patency. The following day, the patient had a formal AN that found no residual stone fragments and confirmed right ureteral patency, confirming the BMBN result, and he subsequently had his nephrostomy tube removed (Figure 1). This case showed how confirmation of ureteral patency with BMBN was possible even in a patient with complex urological anatomy. In the second case, a 64-year-old male underwent right PCNL for a staghorn calculus. He similarly had a 10Fr nephrostomy tube inserted post-operatively and underwent a BMBN that confirmed ureteral patency. He also underwent a formal AN that similarly confirmed ureteral patency (Figure 2).

Methylene blue is a safe, cheap, easily available and approved therapeutic product with multiple well-documented clinical uses including its routine use during PCNL procedures.^2,3.^ Recorded adverse effects include serotonin syndrome risk when combined with other serotonergic drugs, dizziness, confusion, headaches, and urine discoloration.^2^ The “gold standard” antegrade nephrostogram (AN) to assess ureteral patency presents several limitations. Firstly, it is a lengthy investigation, taking at least 90 minutes to perform when including patient transport times. Secondly, it exposes the patient to radiation and contrast agents known to potentially cause serious hypersensitivity reactions. Thirdly, it requires interventional radiology and is therefore reliant on user expertise and experience as well as availability of purpose-specific interventional radiology procedural equipment and staff. Fourthly, it is costly, with a rebatable cost of $164.20 (AUD) according to the Australian Medicare website.^3^ Unlike ANs, BMBNs provide a rapid, low-cost, accurate and real-time evaluation, eliminating the need for patient transport, specialised radiologist equipment and staff, and additional resource costs. With a 5ml vial of methylene blue costing about $26 (AUD) and its rapid test-to-result time of approximately 20 minutes, it allows for a prompt clinical review and optimisation of hospital resources.^4^ For our reported cases, delays in performing the nephrostogram due to contrast and staff shortages led to delays in removing the nephrostomy tubes. This ultimately led to a prolonged hospital stay with its associated additional risks and costs. When projected against the backdrop of costs of hospital stay for PCNL patients, which range from $3,700 to $6,450 AUD, the integration of BMBN in post-operative protocols for PCNL cases may contribute to accelerating the discharge of stable patients, especially when there are no significant risk factors, a low suspicion of significant remaining stone burden and no peri-operative complications.^5^ We therefore propose the attached step-by-step protocol for performing a BMBN in the context of post-operative PCNL patients with a nephrostomy tube.

Conclusion

BMBN is a safe, tolerable, rapid, and cost-effective procedure with no radiation exposure able to confirm ureteral patency following PCNL in patients with a nephrostomy tube. With current bed and staff shortages in public hospitals, scrutinised resource allocation and potential intravenous contrast shortages, BMBN emerges as an alternative method to formal AN to assess ureteral patency. There remains a need for a prospective cohort study or randomised control trial to compare BMBN to formal AN in post-operative PCNL patients, and we present an initial step-by-step protocol of the procedure to assist in this aim.

Figure 1: Methylene blue in the patient’s urine sample (left) suggesting unobstructed antegrade ureteric flow, confirmed by a formal AN where the right nephrostomy tube is first visible (middle) and right ureteral patency is proven by injection of radiopaque contrast (right).


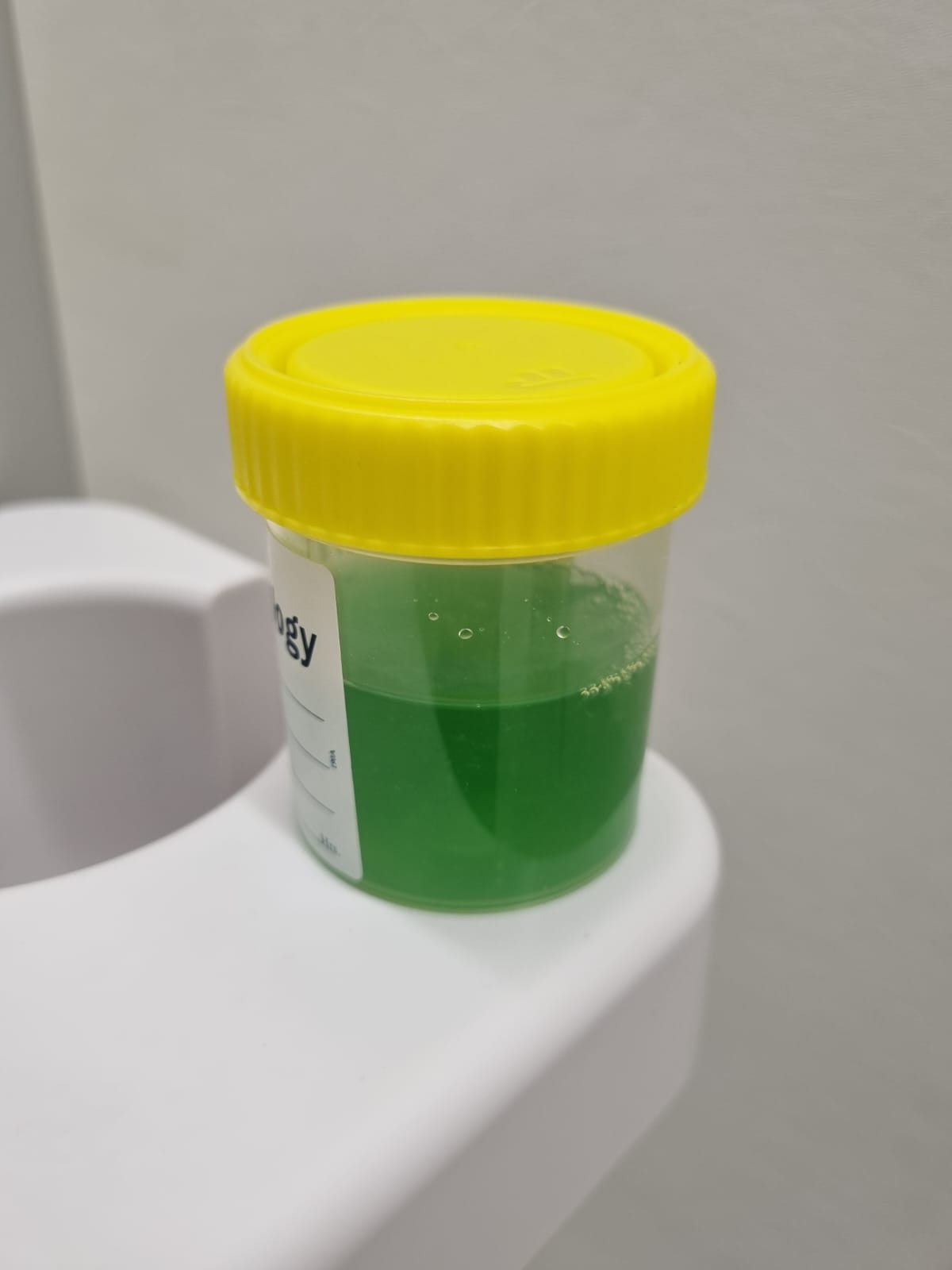

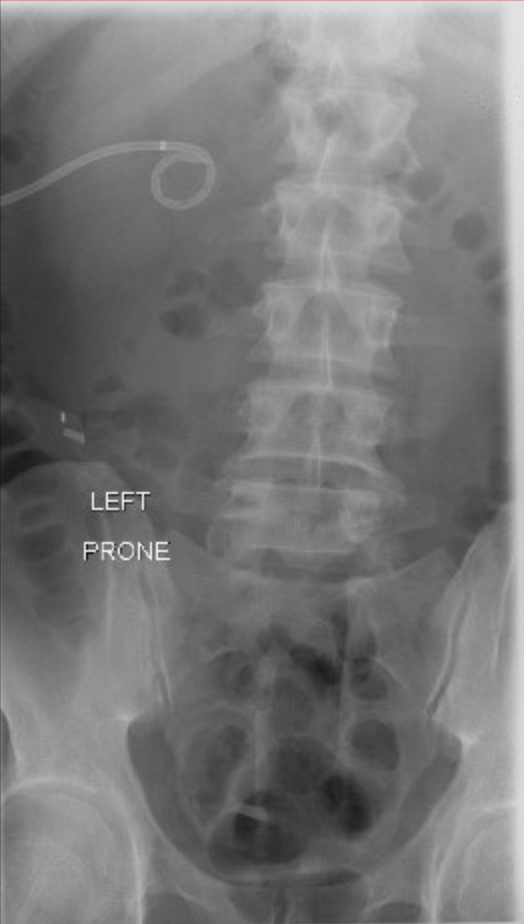

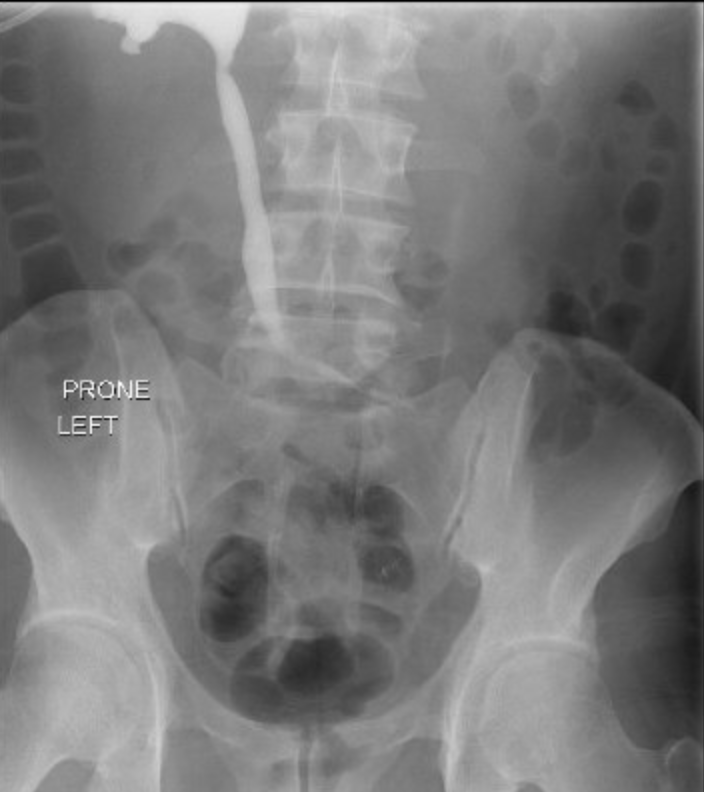


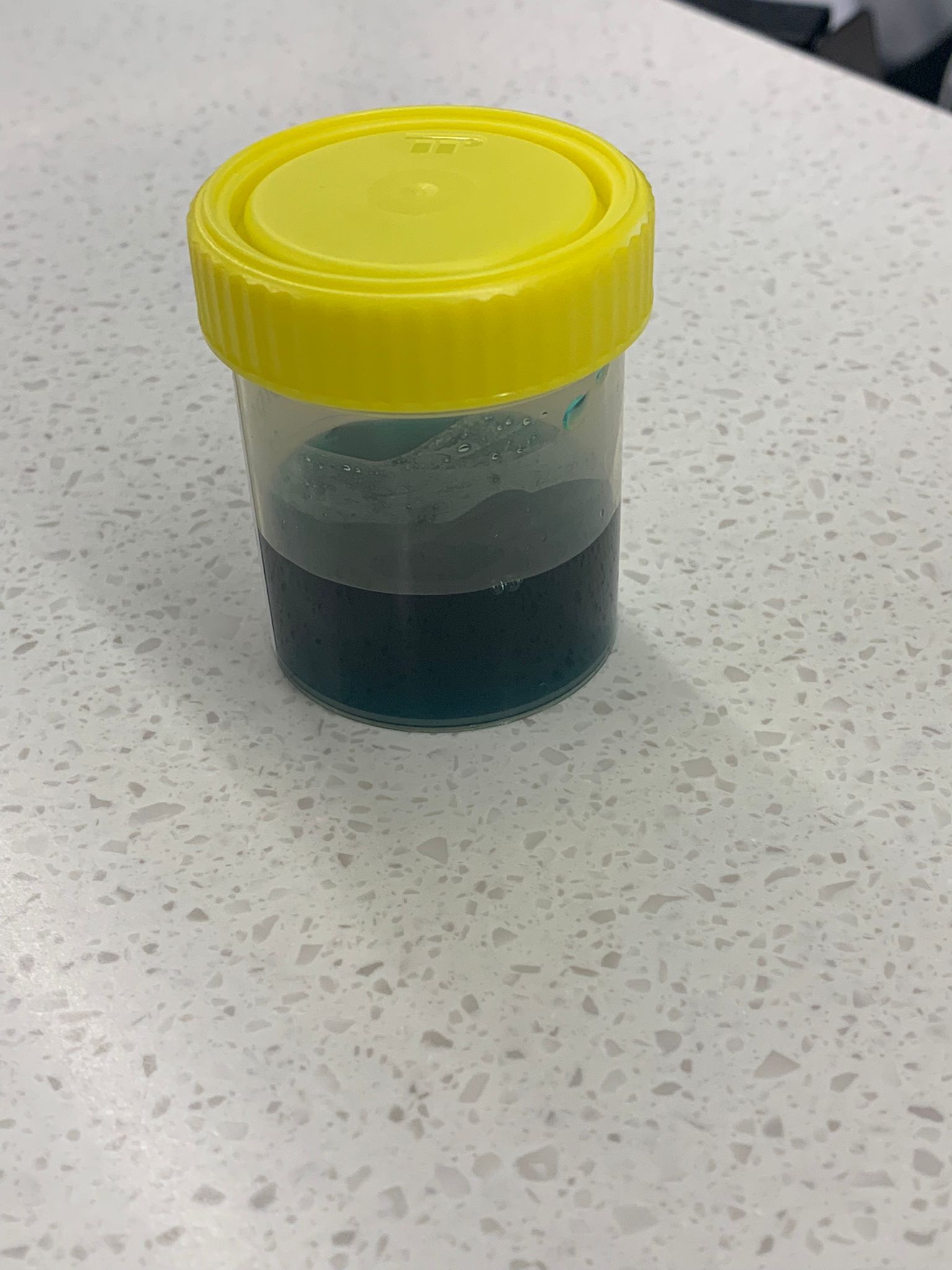

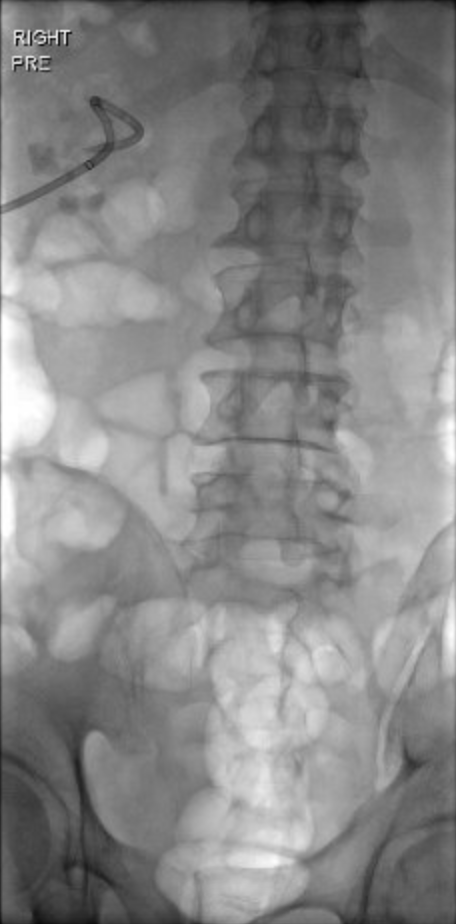

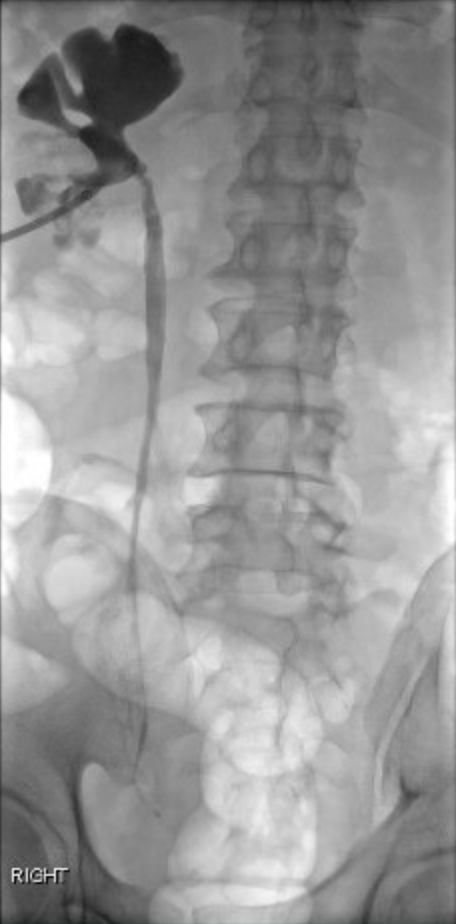


Figure 2: Methylene blue in the patient’s urine sample (left) suggesting unobstructed antegrade ureteric flow, confirmed by a formal AN where the right nephrostomy tube is first visible (middle) and right ureteral patency is proven by injection of radiopaque contrast (right).

References:

Pridgeon S. (2015). Bedside methylene blue 'nephrostogram'. *Annals of the Royal College of Surgeons of England*, *97*(1), 83. <https://doi.org/10.1308/rcsann.2015.97.1.83>

Bistas E, Sanghavi DK. Methylene Blue. [Updated 2023 Jun 26]. In: StatPearls [Internet]. Treasure Island (FL): StatPearls Publishing; 2024 Jan-. Available from: <https://www.ncbi.nlm.nih.gov/books/NBK557593/>

Medicare benefits schedule - item 36650 [Internet]. [cited 2024 Feb 27]. Available from: https://www9.health.gov.au/mbs/fullDisplay.cfm?type=item&q=36650&qt=item

Methylene blue staining solution, concentrated [Internet]. [cited 2024 Feb 27]. Available from: https://www.southernbiological.com/methylene-blue-staining-solution-concentrated/

Hirsch NA. Renal stone therapy, Summary [Internet]. Australian Institute of Health and Welfare. 1991 [Last updated 17/08/2023]. Available from: https://www.aihw.gov.au/reports/chronic-kidney-disease/renal-stone-therapy/contents/summary
